# Deterministic strain-induced arrays of quantum emitters in a two-dimensional semiconductor

**DOI:** 10.1038/ncomms15053

**Published:** 2017-05-22

**Authors:** Artur Branny, Santosh Kumar, Raphaël Proux, Brian D Gerardot

**Affiliations:** 1 Institute of Photonics and Quantum Sciences, SUPA, Heriot-Watt University, Edinburgh EH14 4AS, UK

## Abstract

An outstanding challenge in quantum photonics is scalability, which requires positioning of single quantum emitters in a deterministic fashion. Site positioning progress has been made in established platforms including defects in diamond and self-assembled quantum dots, albeit often with compromised coherence and optical quality. The emergence of single quantum emitters in layered transition metal dichalcogenide semiconductors offers new opportunities to construct a scalable quantum architecture. Here, using nanoscale strain engineering, we deterministically achieve a two-dimensional lattice of quantum emitters in an atomically thin semiconductor. We create point-like strain perturbations in mono- and bi-layer WSe_2_ which locally modify the band-gap, leading to efficient funnelling of excitons towards isolated strain-tuned quantum emitters that exhibit high-purity single photon emission. We achieve near unity emitter creation probability and a mean positioning accuracy of 120±32 nm, which may be improved with further optimization of the nanopillar dimensions.

## Abstract (web summary)

The emergence of quantum emitters in 2D materials has led to the quest for methods and designs enabling their controllable spatial positioning. Here, the authors use strain engineering to fabricate a deterministic array of quantum emitters in WSe_2_ with nanometre positioning accuracy.

Nanoscale strain engineering of the electronic band structure to create quantum confinement has long been pursued in bulk semiconductors. In particular, the exploitation of local elastic strain to laterally confine carriers has been achieved in epitaxially grown heterostructures such as quantum wells^1^,^2^,^3^ or wires^4^. Unfortunately, robust strain-induced quantum confinement of carriers suitably isolated from detrimental surface states in bulk systems has not been realized due to the combination of: (i) the limited elastic strain possible before plastic deformation and (ii) small vertical strain propagation distances. Optically active two-dimensional (2D) semiconductors with a high elastic strain limit^5^ offer renewed opportunities for nanoscale strain engineering of three-dimensional quantum confinement, which we pursue here.

Our approach is to engineer nanoscale strain perturbations in atomically thin WSe_2_. Mono-layer (1L) WSe_2_ is a direct band-gap 2D semiconductor with a dark excitonic ground state^6^,^7^. In this system, at sufficiently low temperatures, a dense ensemble of localized excitons emerges at lower energy than the broad (few meV) delocalized 2D excitons^8^,^9^,^10^. While their precise nature is not fully understood, the optically bright localized excitons are possibly related to disorder, scattering or crystal imperfections^11^,^12^. Under certain conditions, such as at flake interfaces or edges^13^,^14^ or at local strain pockets due to flake wrinkling, folding or bubbling^15^,^16^,^17^,^18^,^19^, individual localized excitons with narrow linewidths (sub-100 micro-eV) can be isolated and shown to emit single photons^13^,^14^,^15^,^16^,^17^,^18^,^19^,^20^,^21^,^22^,^23^.

Here we exploit the correlation of spectrally and spatially isolated single quantum emitters and point-like strain perturbations^16^ to engineer quantum emitter arrays in a deterministic fashion. Figure 1 shows a sketch of the concept. Owing to Van der Waals' forces a 2D flake conforms to the contours of a lithographically patterned nanopillar lattice (Fig. 1a), inducing significant elastic, point-like strain at the nanopillar lattice points^24^,^25^. The bi-axial strain at the nanopillar locations locally modifies the band-gap and spatially modulates the potential landscape of the 2D excitons (Fig. 1b). As a result, photogenerated excitons funnel from larger band-gap regions (in between the nanopillars) into smaller band-gap regions (nanopillar centres)^25^,^26^,^27^. Superimposed onto the artificially induced potential energy landscape are a high density of randomly distributed localized excitons, shown in Fig. 1b as small perturbations with quantized energy levels. In the unstrained regions (between the nanopillars), the localized excitons overlap in energy and cannot be distinguished. However, with the appropriate nanopillar dimensions and localized exciton density, an individual localized exciton will be strain tuned at each lattice site. The strain tuned quantum emitters at the nanopillar locations will dominate the photoluminescence (PL) spectrum at low temperature and excitation power due to efficient exciton funnelling from the surrounding high band-gap regions to the single lowest energy localized exciton.

We implement this concept to achieve arrays of robust quantum emitters in both mono- and bi-layer (2L) WSe_2_. In addition, by varying the aspect ratio of the lithographically patterned nanopillar arrays we are able to fully characterize the technique and optimize the positioning accuracy of the localized excitons, ultimately achieving an accuracy of 120±32 nm with near unity yield. This precision can likely be further improved by refining the nanopillar geometry and dimensions.

## Results

### Fabrication and structural characterization

To achieve point-like strain perturbations in atomically thin WSe_2_, we use an all-dry transfer technique^28^ to place mechanically exfoliated flakes onto a substrate with a square lattice (4 μm pitch) of dielectric nanopillars fabricated by electron beam lithography. This technique takes advantage of Van der Waals' forces to ensure the 2D flake conforms to the contours of the patterned substrate and induces significant elastic strain at the locations of the nanopillars. A WSe_2_ flake consisting of 1L and 2L regions is shown in Fig. 2a,b before and after transfer, respectively. In Fig. 2b the nanopillars are identified by the change in contrast: the dark points in the micrograph correspond to locations of the nanopillars. The transferred flake's topography is characterized by atomic force microscopy (AFM), as shown in Fig. 2c,d. Scanning electron imaging of the same region (Supplementary Fig. 1) confirms the same physical features. Figure 2d compares the cross-section of a bare nanopillar (#0 in Fig. 2c) with an aspect ratio (height to width) of ∼0.3 to that of a flake over a nanopillar (#7 in Fig. 2c). Ideally, the flake conforms to the nanopillar without significant wrinkling as it does in #7. At other lattice positions, the flake does not conform closely to the substrate topography but rather stretches over the nanopillar analogously to a canvas over a tent-pole. Randomly oriented pleats emerging from nanopillars are observed as well as ripples which in some cases extend towards neighbouring nanopillars. These features are typical of 2D flakes suspended over corrugated substrates and can be further engineered^24^,^25^. Importantly, for the 4 μm pitch array used here, the wrinkles do not mask the point-like strain perturbation created by the nanopillars.

### A monolayer WSe_2_ quantum emitter array

Hyperspectral confocal PL imaging is performed to fully characterize the atomically thin WSe_2_ flake and the effects of strain perturbations. Figure 2f shows a colour-coded spatial map of the peak intensity of the PL spectrum (660–830 nm). The PL peak (integrated) intensity significantly increases by a factor of 50 (2) at the locations of the nanopillars, likely due to efficient diffusion of excitons towards the lower energy states at the strain-tuned sites^25^,^26^,^27^. The spectra from several nanopillar sites as well as typical 1L and 2L regions between the nanopillars are exhibited in Fig. 2g. A broad defect band with weak intensity is observed in the homogeneous 1L and 2L regions (at low excitation powers the 2D excitons are not visible). On the other hand, for each spectrum obtained at a location of a nanopillar (including nanopillar #1 in the 2L region), a few discrete narrow linewidth (<200 μeV) peaks with high intensity are typically observed. Each of these peaks signify emission from single quantum emitters^13^,^14^,^15^,^16^,^17^,^18^,^20^,^21^,^22^. A second-order correlation measurement is presented in Fig. 2e, where a fit to the data yields *g*^(2)^(0)=0.07±0.04 with a decay time of *τ*=2.8±0.2 ns. This demonstrates photon antibunching and the quantum nature of the 1L discrete lines.

### A bilayer WSe_2_ quantum emitter array

To demonstrate the universality of nanoscale strain engineering to generate strain-induced quantum emitters in atomically thin semiconductors, we create an array of pure single photon emitters in 2L WSe_2_. Bi-layer WSe_2_ is an intriguing host for quantum emitters as it offers an additional pseudo-spin based on the layer degree of freedom^29^. While 2L WSe_2_ is an intrinsically indirect band-gap semiconductor, the indirect and direct transitions are nearly degenerate and under tensile strain the band structure can be modified such that the direct transition becomes preferred^30^. We exfoliate a flake with a large 2L region (see Fig. 3a) and transfer it to an array of nanopillars (with h:w ∼0.3) on an Si substrate (Fig. 3b). Once again, we observe a significant increase of 150 × (3 × ) in the peak (integrated) PL intensity at the nanopillar sites (Fig. 3c), evidencing the transition to a direct electronic gap and the exciton funnel effect due to local strain. We note there is also a small 1L and a large 3L region of the flake in Fig. 3b that cover nanopillar sites. While the 1L region shows similar properties to the flake in Fig. 2, the 3L remains dark in PL. Crucially, bright narrow-linewidth spectral lines (Fig. 3h) are again observed at the strain-induced sites. Figure 3d shows a high-resolution spatial map of six nanopillars in the centre of the array superimposed with the locations of the quantum emitters in Fig. 3h. A wavelength histogram (2 nm binning) of all strain-induced emitters (53 in total) created in the 2L WSe_2_ array is shown in Fig. 3e. The histogram shows that emitters span a wavelength region from 775 to 835 nm. A Gaussian fit to the data is used to quantify the inhomogeneous distribution of the emitters, yielding 33 meV full width at half maximum (FWHM). Notably, several emitters at very similar wavelengths are observed (for example, in Fig. 3h peaks at 793 nm in spectra 2 and 5; peaks at 785 nm in spectra 4 and 6). The second-order correlations from each individual peak measured exhibit highly pure single photon emission, for example, the single peak at *λ*=801.08 nm from nanopillar #1 in Fig. 2g shows *g*^(2)^(0)=0.03±0.02 with a decay time of *τ*=4.8±0.1 ns (Fig. 3f). For spectra recorded over a 20-h period, this emitter does not blink. Figure 3g shows a histogram of the spectral jitter recorded over 20 h (using 3 s time-bin, as shown in Supplementary Fig. 2) from the 2L emitter at nanopillar #1. The histogram is fit by a Gaussian distribution with 131 μeV FWHM. Further, these quantum emitter arrays are optically stable and robust, surviving multiple sample cooling and heating cycles.

### Optimization of strain-induced arrays of quantum emitters

Having established a technique to successfully create robust strain-induced quantum emitters, we seek to optimize the process. We tailor the local elastic strain by varying the height to change the aspect ratio of five rows of nanopillars, from h:w 0.15 to 0.59, as shown in the scanning electron micrograph in Fig. 4a. Further information on these nanopillars is provided in Supplementary Fig. 3. Precise measurements of the pillar size are made by AFM which confirms uniform pillar dimensions within each row. Their cross-sections are displayed in Fig. 4c. Figure 4b shows an optical micrograph of a large (∼100 × 25 μm) 1L WSe_2_ flake covering 101 nanopillars in the array. Here the pillar locations are identified by the bright points in the micrograph. Also visible in Fig. 4b in some cases for the high aspect ratio nanopillars (for example, Row 5, Columns 3–5) is a small ring surrounding the pillar. This feature is not observed for any of the low aspect ratio nanopillars in Rows 1 and 2 or with the nanopillars used in Figs 2 and 3. Figure 4d,e shows a spatial map of the integrated and peak intensities (logarithmic scale) of the entire PL spectrum (690–850 nm) from Columns 2 to 5 (as labelled in Fig. 4b). Mapping the peak intensity reveals the locations of the intense, narrow linewidth peaks that signify quantum light emission. PL maps of the entire flake with several spectra are shown in Supplementary Fig. 4. A mixture of peaks with large (700–900 μeV) fine-structure splittings (for example, Supplementary Fig. 5) and single linearly polarized peaks are observed, as reported previously^18^. The PL intensity at the nanopillars increases with increasing nanopillar aspect ratio, as expected for the diffusion of excitons towards the local regions with strain-tuned band-gaps. In the cases for the high aspect ratio nanopillars where the ring surrounding the pillar is visible in Fig. 4b (for example, Row 5, Columns 3–5), low intensity emission at the nanopillar centre is surrounded by a ring of intense PL. We believe this effect is caused by the flake being pierced during the transfer process for some high aspect ratio nanopillars. Alternatively, the flake could conform quite closely to the nanopillar topography and sit flat at the top and then bend at the top edge of the nanopillar, leading to high strain at the circumference.

Statistics of all emitters identified (285 in total) in the entire 1L WSe_2_ flake over the 101 nanopillars is shown in Fig. 4f,g. Here we do not account for the possibility that multiple emission lines arise from a single emitter (for example, due to different charge states or bi-excitons^23^) and assume that each narrow linewidth peak in a spectrum is an individual quantum emitter. Second-order correlation measurements on numerous narrow linewidth peaks yield *g*^(2)^(0)<0.5, unambiguously demonstrating the quantum nature of the emission. Figure 4f shows the statistics for the number of emitters per pillar. While distinct emitters are sometimes found at the nanopillar sites in Row 1 (for example, the spectra in Supplementary Fig. 4c), they tend to be difficult to distinguish from the broad background. With the increased aspect ratio of the nanopillars in Row 2, one or two distinct bright emitters are found with a yield of 85% (17 of 20 nanopillars contain pure single emitters). Rows 3 and 4 have near unity yields: 96% (45 of 47 nanopillars yield at least 1 quantum emitter).

Figure 4g shows the emitter wavelength histogram. While the overall energy distribution of the single emitters is broad (spanning ∼200 meV), all emitters emit at lower energy than the bright 2D exciton peak in WSe_2_ (∼1.74 eV). The emitter wavelength histogram is also post-selected for emitter brightness (the peak intensity at ∼0.8 saturation power) for this flake. Fitting a Gaussian distribution to the histogram gives 73 meV FWHM, more than twice as large as the 2L emitters.

### Positioning accuracy

To determine the positioning accuracy of the strain-induced quantum emitters we map the emitter locations relative to the centre of the nanopillar. While the individual emitters are resolved by the hyperspectral spatial maps (see Figs 4e and 5c) the precise position of the nanopillars is not known *a priori* but can be extracted using spectral weighted averaging (WA_*λ*_), as illustrated in Fig. 5. WA_*λ*_ accounts for the global shift in the PL spectrum due to the strain, as can be observed in two representative spectra from the centre of the nanopillar and the nominally unstrained region between the nanopillars shown in Fig. 5a. At each spatial location, we quantify this spectral shift using the formula:


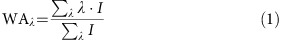


The outcome yields smooth, clean contour maps, as shown in Fig. 5b for nanopillars from Column 5 and Rows 2–5. Assuming the strain is at its highest in the middle of the nanopillar, we fit Gaussian curves to the WA_*λ*_ cross-sections to determine the centre of each nanopillar with high accuracy. Further, a rough estimate of the strain can be based on the spectral shift^30^. For example, a maximum change in WA_*λ*_=30.8 meV (13.3 nm) for nanopillar 4 corresponds to 0.60% strain. This value is three times larger than that previously reported for local strain pockets^16^. Finally, in order to obtain the emitter displacement, we distill the locations of the single quantum emitters from the peak intensity maps (for example, as in Fig. 5c) and overlap them with the centres of the nanopillars determined by the Gaussian fits to WA_*λ*_, as shown in Fig. 5d for Column 5. To assess the accuracy of emitter positioning we combine emitter displacements from Columns 2 to 5 and arrive with distributions as displayed in Fig. 5e. Here the red rings represent the mean distance and the ring thickness corresponds to one standard deviation.

We observe that when the flake is pierced or bends over the circular edge of a flat-topped nanopillar, the emitters are found at the circumference of the nanopillar. On the other hand, the quantum emitters are created in the centre of the nanopillars for flakes that conform to the substrate topography to create a point-like strain perturbation. In particular, the statistics of the positioning accuracy of the emitters in Row 2 reveals high precision: 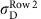
=120±32 nm, where *σ*_D_ is the average displacement of the emitter from the nanopillar centre. Displacement statistics of the emitters in Rows 3–5 are: 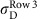
=262±46 nm, 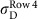
=476±85 nm and 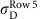
=521±64 nm. Row 1 did not yield sufficient emitter numbers for useful statistics. In the best case (Column 5, Row 2), the displacement is 30 nm.

## Discussion

Layered transition metal dichalcogenide semiconductors are attractive hosts for quantum emitters due to the unique valley degree of freedom and strong spin-orbit coupling. Using nanoscale elastic strain engineering, we have achieved local modification of the electronic and optical properties to deterministically generate robust quantum emitters in this emerging quantum photonic platform. The straightforward fabrication procedure presented here is scalable and likely to be applicable to other 2D materials. We obtain the best results, including near unity success in generating an emitter at each nanopillar and a positioning accuracy of 120 nm, using nanopillars with 0.3 aspect ratio. Further improvement in the positioning accuracy of single emitters is likely possible with smaller diameter nanopillars that maintain an aspect ratio similar to 0.3. Notably, we observe negligible background signal at the base of the discrete spectral lines, enabling high purity single photon emission in both 1L and 2L WSe_2_. Compared to mono-layer WSe_2_, the bi-layer appears additionally attractive as a host for single excitons due to a narrower inhomogeneous distribution and the potential for spin-layer locking. These results open a path to deterministically embed 2D quantum emitters in electronic devices for further investigation of their spin, electronic and optical properties or to couple to cavity or waveguide modes for cavity quantum electrodynamics experiments and integrated quantum photonics applications^31^,^32^.

## Methods

### Sample fabrication

**Figure 1:**
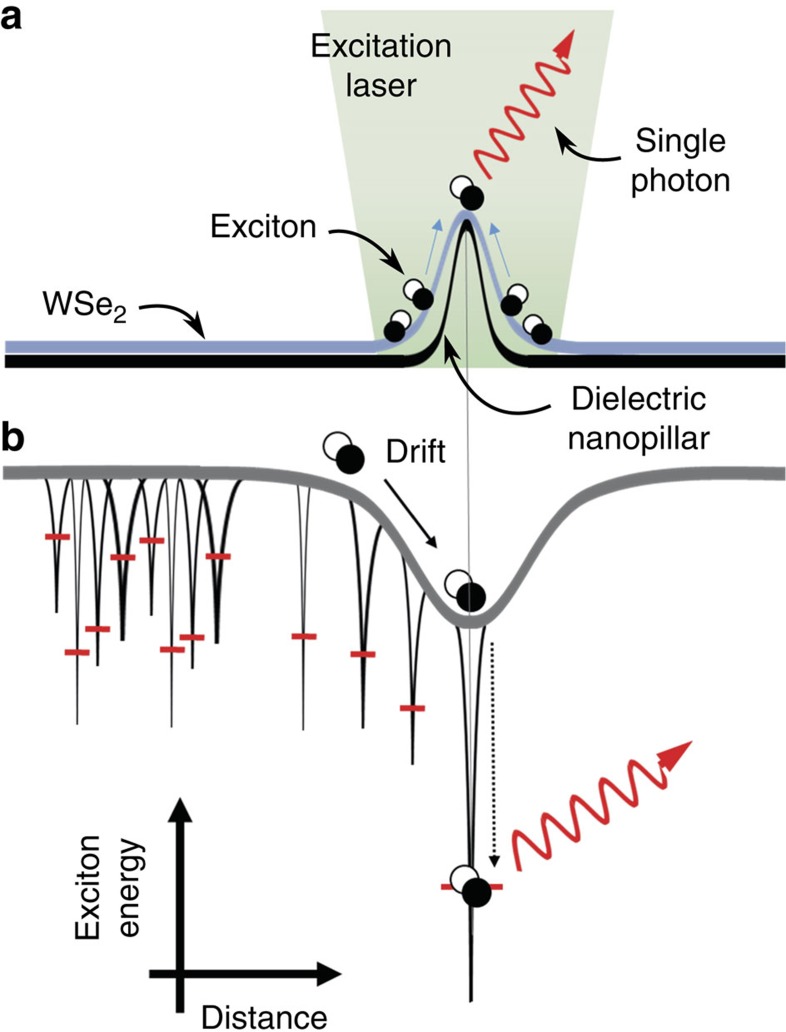
A diagram of the scheme to obtain a strain-induced quantum emitter. (**a**) Atomically thin WSe_2_ is deformed by a nanopillar to achieve a point-like elastic strain perturbation. (**b**) The strain locally modulates the WSe_2_ band-gap. Superimposed on this artificial modulation of the exciton energy are randomly distributed localized excitons. Optically created excitons efficiently funnel to an individual strain tuned localized exciton trap at the nanopillar centre resulting in a single highly efficient quantum emitter.

**Figure 2:**
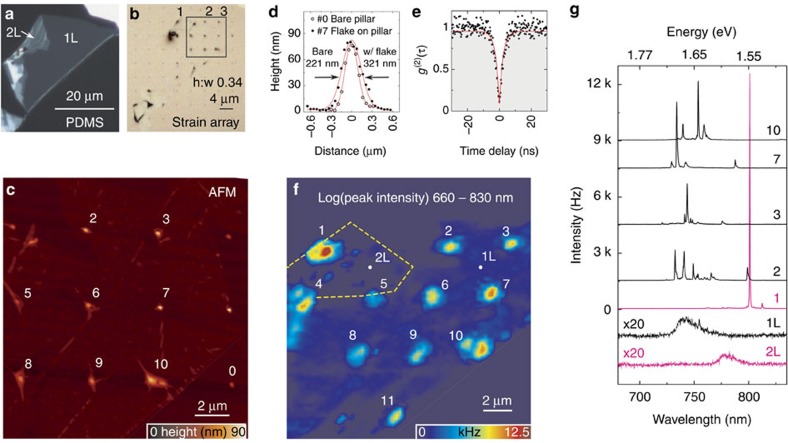
A monolayer WSe_2_ quantum emitter array. Optical micrographs of an exfoliated 1L WSe_2_ flake (**a**) before and (**b**) after transfer onto an Si substrate with an array of dielectric nanopillars. The black box in **b** identifies the region shown in **c** and **f**. (**c**) AFM image of the topography of the flake on top of the nanopillars, revealing a lattice of locally strained points. (**d**) Cross-section AFM profile of a bare nanopillar #0 and nanopillar #7 that is covered by the monolayer. (**e**) Second-order photon correlation statistics from a typical 1L quantum emitter revealing clear antibunching [*g*^(2)^(0)=0.07±0.04 and *τ*=2.8±0.2 ns]. (**f**) Colour-coded spatial map of the peak PL signal in the spectral range of 660–830 nm. (**g**) Example PL spectra of isolated quantum emitters at the pillar locations as labelled. Also shown is the weak signal from the unstrained 1L and 2L WSe_2_.

**Figure 3:**
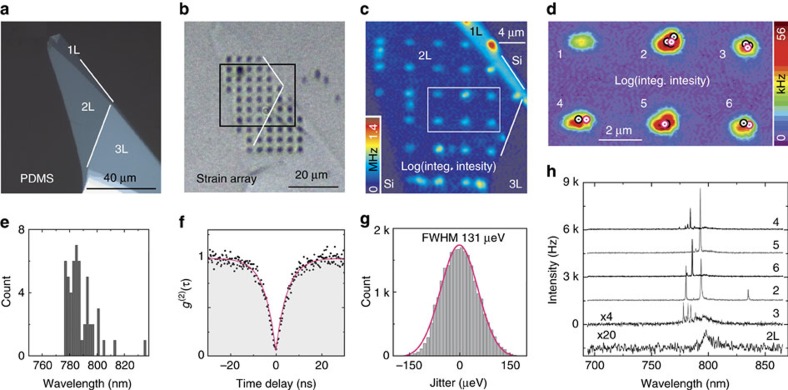
A bilayer WSe_2_ quantum emitter array. Optical micrograph of 2L WSe_2_ (**a**) before and (**b**) after the transfer onto the nanopillars. (**c**) A 2D spatial map of the PL integrated intensity within 700–860 nm. (**d**) A high resolution spatial map of integrated intensity of the six nanopillars indicated in **c**. The red circles mark the positions of emitters in **h**. (**e**) A histogram of the wavelengths of the 2L WSe_2_ emitters. (**f**) Photon auto-correlation histogram from the bi-layer emitter at nanopillar #1 of Fig. 1. A fit using *g*^(2)^(0)=0.03±0.02 and *τ*=4.8±0.1 ns is shown. (**g**) A histogram of the spectral jitter over 20 h (3 s time-bin) of the emitter at nanopillar #1 of Fig. 1. (**h**) PL spectra from the emitter positions identified in **d** with red circles.

**Figure 4:**
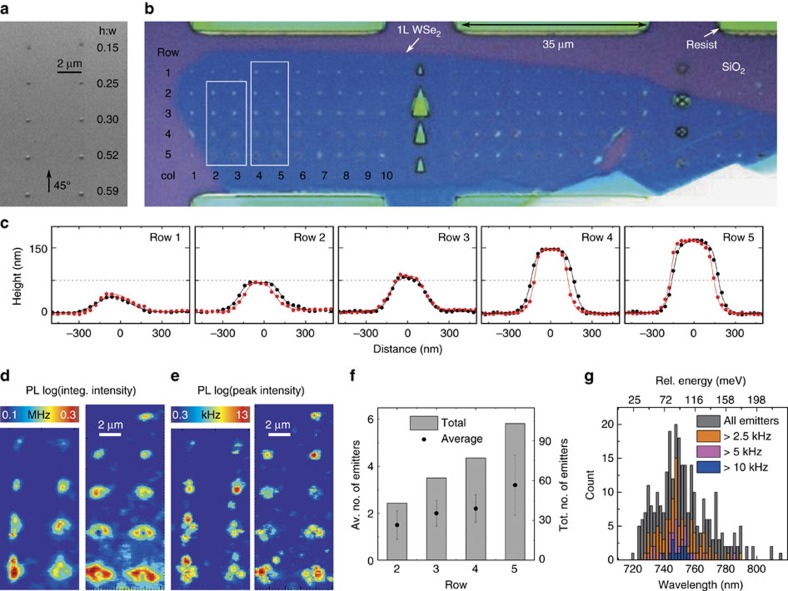
Optimization of strain-induced arrays of quantum emitters. (**a**) A 45° SEM image of an SiO_2_ substrate with an array of nanopillars of varying aspect ratios, as labelled. (**b**) Optical micrograph of a large (100 × 25 mm) 1L WSe_2_ flake covering 101 nanopillars. Some high aspect ratio nanopillars exhibit a dark centre with a bright ring, which suggests the pillar punctured the flake during the transfer process. (**c**) Cross-section AFM profiles of bare nanopillars from different rows with varying aspect ratio. (**d**) High-resolution colour-coded spatial map of integrated PL signal in the spectral range of 690–850 nm for columns 2–5 from the region outlined in **b**. For the flake at nanopillars with higher aspect ratios there is a clear ring in the PL intensity. (**e**) Same maps as **d** showing the peak intensity where the individual emitters are resolved. (**f**) Statistics on the emitters per pillar for each row where each error bar represents a single standard deviation from the mean. (**g**) Histogram of the emitter wavelengths with post-selection on the emitter brightness (peak intensity at 0.8 saturation level).

**Figure 5:**
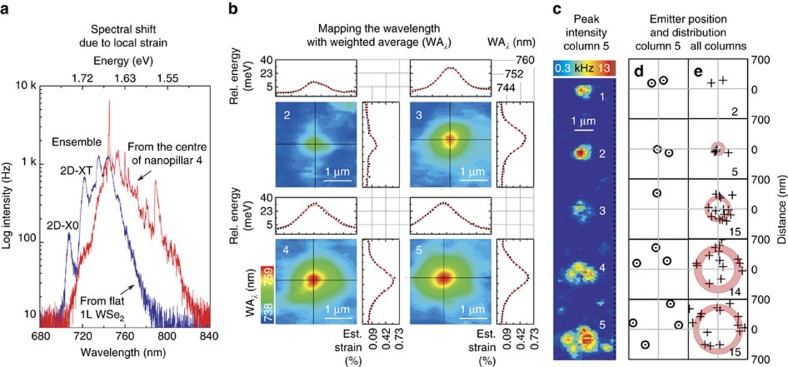
Positioning accuracy determined by weighted spectral averaging. (**a**) Typical PL spectra from the bi-axially strained WSe_2_ monolayer at the centre of a nanopillar (red spectrum) and an unstrained region (blue spectrum). 2D-X0 (2D-XT) refers to the non-localized neutral (charged) exciton state. (**b**) The colour-coded spatial maps of weighted average wavelength (WA_*λ*_) for pillars 2–5 from **c**. The nanopillar centre positions are determined by fitting Gaussian to the WA_*λ*_ profile, shown by the cross-sections. (**c**) The high-resolution colour-coded PL spatial map of peak intensity from column 5. The individual emitters are resolved and can be compared against the WA_*λ*_ mapping. (**d**) Positions of single emitters (black circles) relative to the centre of the nanopillars (grey crosses) for each row from column 5. (**e**) Positions of all emitters (black crosses) in columns 2–5. The red rings represent the average emitter displacement and the ring thickness represents the standard deviation. The number of emitters mapped is identified in the lower right corner of each emitter location map.

We fabricate the nanopillars out of negative resist (AZ 2070 from MicroChemicals) using electron beam lithography. First the resist was spin-coated (∼200 nm thickness) onto the Si substrate (for the samples in Figs 1 and 2) or an Si substrate with 285 nm SiO_2_ (for the sample in Fig. 3) and then exposed using electron beam lithography (Raith Pioneer). For Samples 1 and 2 a constant dose was used for all nanopillars. For Sample 3 the dose was varied but the pillar size was kept constant. The nanopillars were then developed (AZ 726 MIF Developer from Electronic Materials) and structurally characterized by SEM and AFM. Mechanical exfoliation was used to obtain mono- or bi-layer WSe_2_ flakes which are identified by optical microscopy. The chosen flakes were then transferred to the nanopillars using the all-dry viscoelastic stamping procedure outlined in ref. 28. Each flake's thickness is then confirmed by low-temperature PL.

### Low-temperature confocal photoluminescence

A home-made confocal microscope with an objective lens (NA of 0.82 yielding a diffraction limited focus of ∼560 nm at *λ*=750 nm) is used to spatially map the PL from the sample which is placed on automated nanopositioners in a *T*=3.5 K closed-cycle cryostat. All spectra were acquired with a 0.5 m focal length spectrometer and a nitrogen-cooled charge-coupled device with a measured spectral resolution of ∼75 μeV at *λ*=750 nm for an 1,800 l mm^−1^ grating. A fibre-based Hanbury-Brown and Twiss interferometer was used for second-order correlation measurements, and photon counting was performed using Si APDs.

### Data availability

The data that support the findings of this study are available from the corresponding author on request.

## Additional information

**How to cite this article:** Branny, A. *et al*. Deterministic strain-induced arrays of quantum emitters in a two-dimensional semiconductor. *Nat. Commun.* **8,** 15053 doi: 10.1038/ncomms15053 (2017).

**Publisher's note**: Springer Nature remains neutral with regard to jurisdictional claims in published maps and institutional affiliations.

## Supplementary Material

Supplementary Information Supplementary Figures and Supplementary Notes

Peer Review File
