# Supplementary material for: Deterministic strain-induced arrays of quantum emitters in a two-dimensional semiconductor
Source: Nat Commun. 2017 May 22;8:15053. doi: 10.1038/ncomms15053 (PMC5458118; doi:10.1038/ncomms15053)
Supplement: Supplementary Information — Supplementary Figures and Supplementary Notes [file ncomms15053-s1.pdf]

Supplementary Note 1. Structure of monolayer WSe_2 strain array.

Supplementary Figure 1 shows scanning electron microscope (SEM) images of a monolayer WSe_2 transferred onto the nanopillars. The topography observed here matches that observed in the atomic force microscope (AFM) image of Fig. 1c in the main article. A magnification of the areas framed in Supplementary Figure 1a is presented in Supplementary Figure 1b, c and d. The wrinkled monolayer around nanopillar 6 is clearly visible in Supplementary Figure 1c. Supplementary Figure 1d shows a bare nanopillar for comparison (identified by a white square at the bottom right of Supplementary Figure 1).

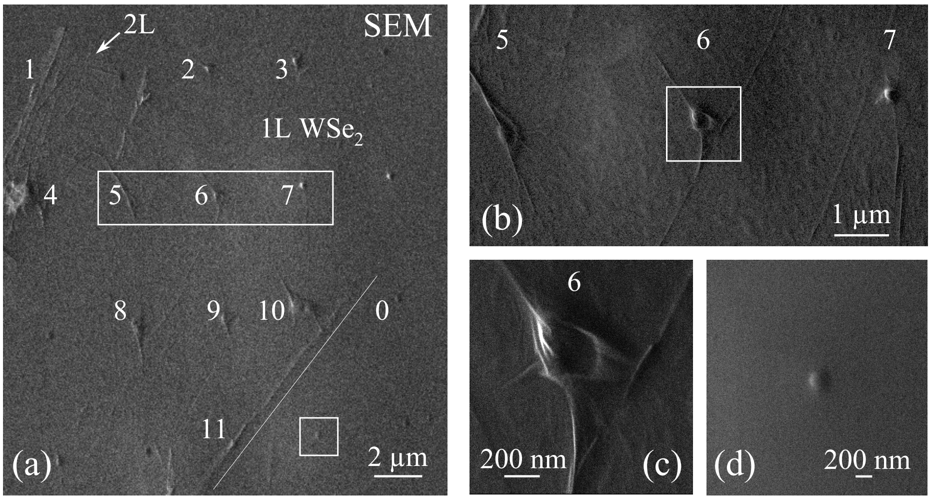

Supplementary Figure 1. Monolayer WSe_2 strain array under scanning electron microscopy (SEM). SEM images of the monolayer WSe_2 flake on nanopillars presented in Fig. 1 of main article. (a) The entire flake with the region of interest. (b) Magnification of nanopillars 5, 6 and 7. (c) Magnification of nanopillar 6. The wrinkling of the monolayer at the nanopillar correlates with that measured by AFM in the main manuscript. (d) A bare nanopillar is shown for comparison. It is identified at the bottom right of (a) by a white square.

Supplementary Note 2. Stability of bilayer WSe_2 quantum emitter.

Supplementary Figure 2 characterizes more precisely the 2L emitter 1 of Fig. 1 of the main article. Supplementary Figure 2a shows a time-trace of the peak energy over 20 hours of measurement. The standard deviation is 53 μeV), showing good emission energy stability. Supplementary Figure 2b is an intensity histogram performed on the same time-trace measurement. It accounts for an excellent intensity stability with no blinking observed. The majority of intensity fluctuations are due to vibrations during the period. Finally, Supplementary Figure 2 is a power dependence of the integrated photoluminescence intensity. The emitter exhibits a very clear saturation behaviour consistent with a two-level system.

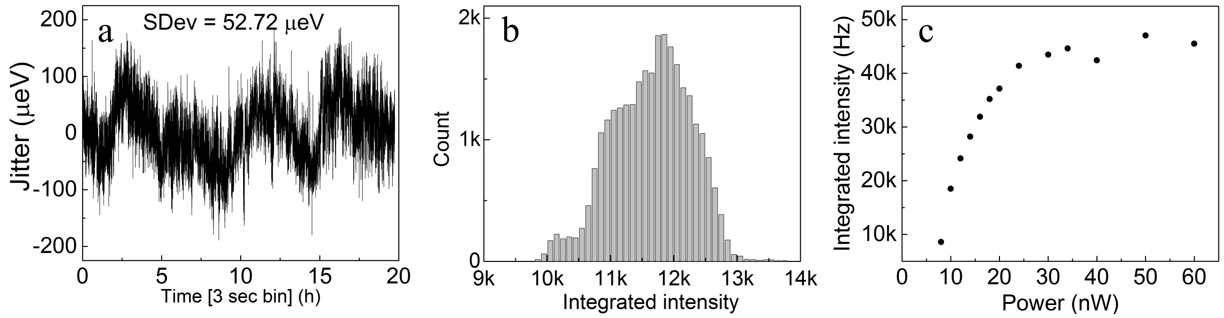

Supplementary Figure 2. In-depth optical characterization of bilayer WSe_2 quantum emitter. (a) Time-trace of the peak energy detuning of the photoluminescence of emitter 1 in Fig. 1 in a bilayer flake of WSe_2, using 3 s time bins. (b) Histogram of intensity performed on the previous time-trace. (c) Power dependence of the integrated intensity emitted by this emitter, showing a typical saturation behaviour.

Supplementary Note 3. Nanopillar array with varying dimensions.

Supplementary Figure 3 shows the nanopillars characterization on the same sample as Fig. 4, prior to the deposition of the monolayer WSe_2. Supplementary Figure 3a are vertical profiles of the nanopillars for each row. Supplementary Figure 3b displays the dimensions and aspect ratio extracted from Supplementary Figure 3a. The width stays constant at 280 ± 20 nm for all rows while the height increases from 40 nm to 170 nm. Supplementary Figure 3c is an SEM image of the sample, where the different rows of nanopillars are visible corresponding to different aspect ratios. Supplementary Figure 3d is a magnification of nanopillars from rows 2, 3 and 4. The change in height is clearly visible.

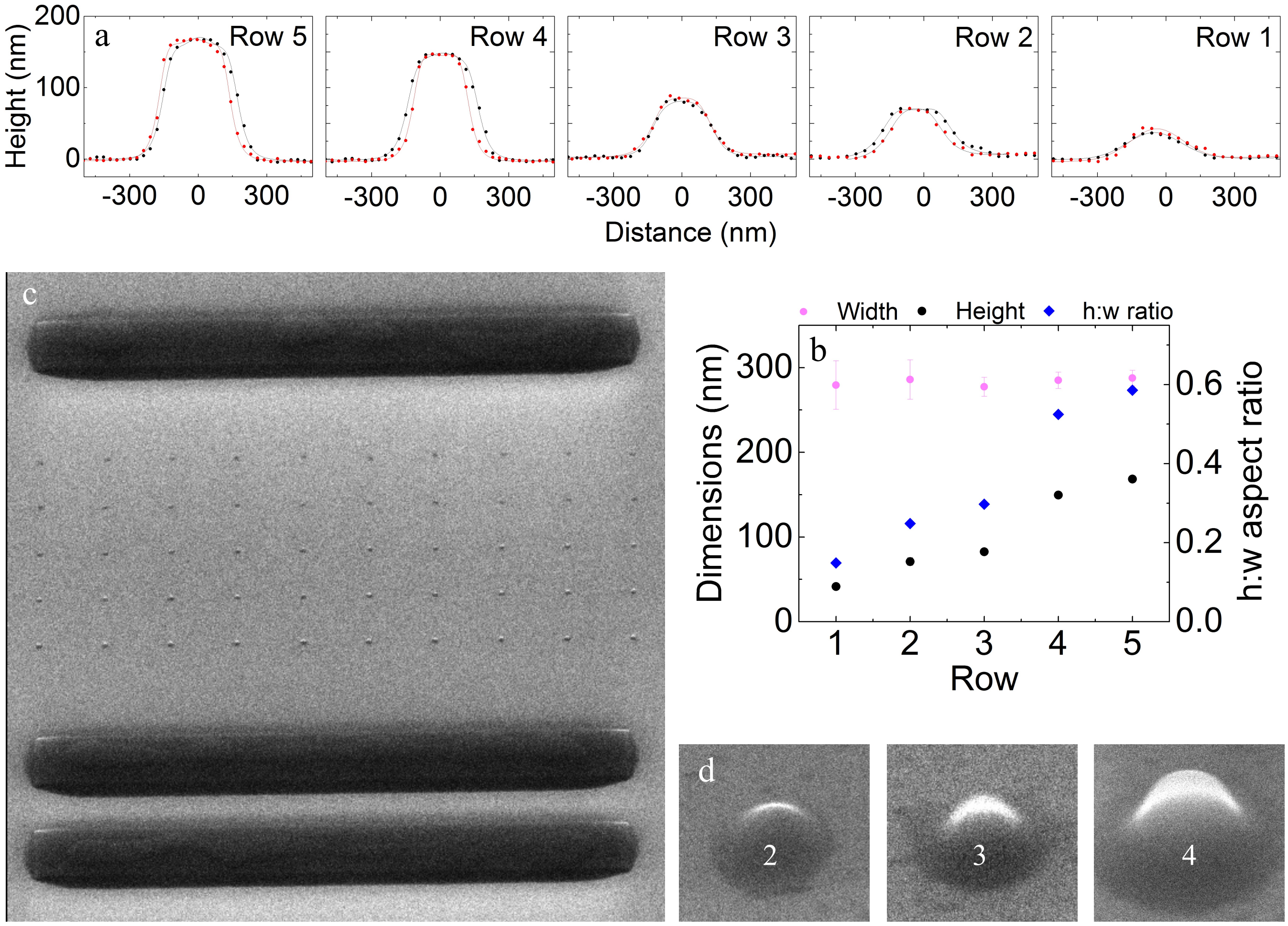

Supplementary Figure 3. Nanopillar structural characterization. (a) AFM profile of nanopillars for each row of the sample shown in Fig. 3 of the main article and 4. (b) Dimensions and aspect ratio of the nanopillars for each row, calculated from (a). (c) SEM image of the sample showing nanopillars of different heights for each row. (d) Detailed SEM image of nanopillars in rows 2, 3 and 4, respectively with aspect ratios 0.25, 0.30 and 0.52.

Supplementary Note 4. Photoluminescence of large area array of single quantum emitters.

Supplementary Figure 4 shows more extensive data on the monolayer WSe_2 sample displayed in Fig. 3 of the main article. Supplementary Figure 4a shows the larger view of the same optical micrograph as Fig. 3b. Supplementary Figure 4b shows 4 different spatial maps stitched together displaying the integrated photoluminescence intensity on the color scale. PL intensity is systematically enhanced at the position of the nanopillars. Supplementary Figure 4c shows several photoluminescence spectra for diverse quantum emitters on the position of the nanopillars.

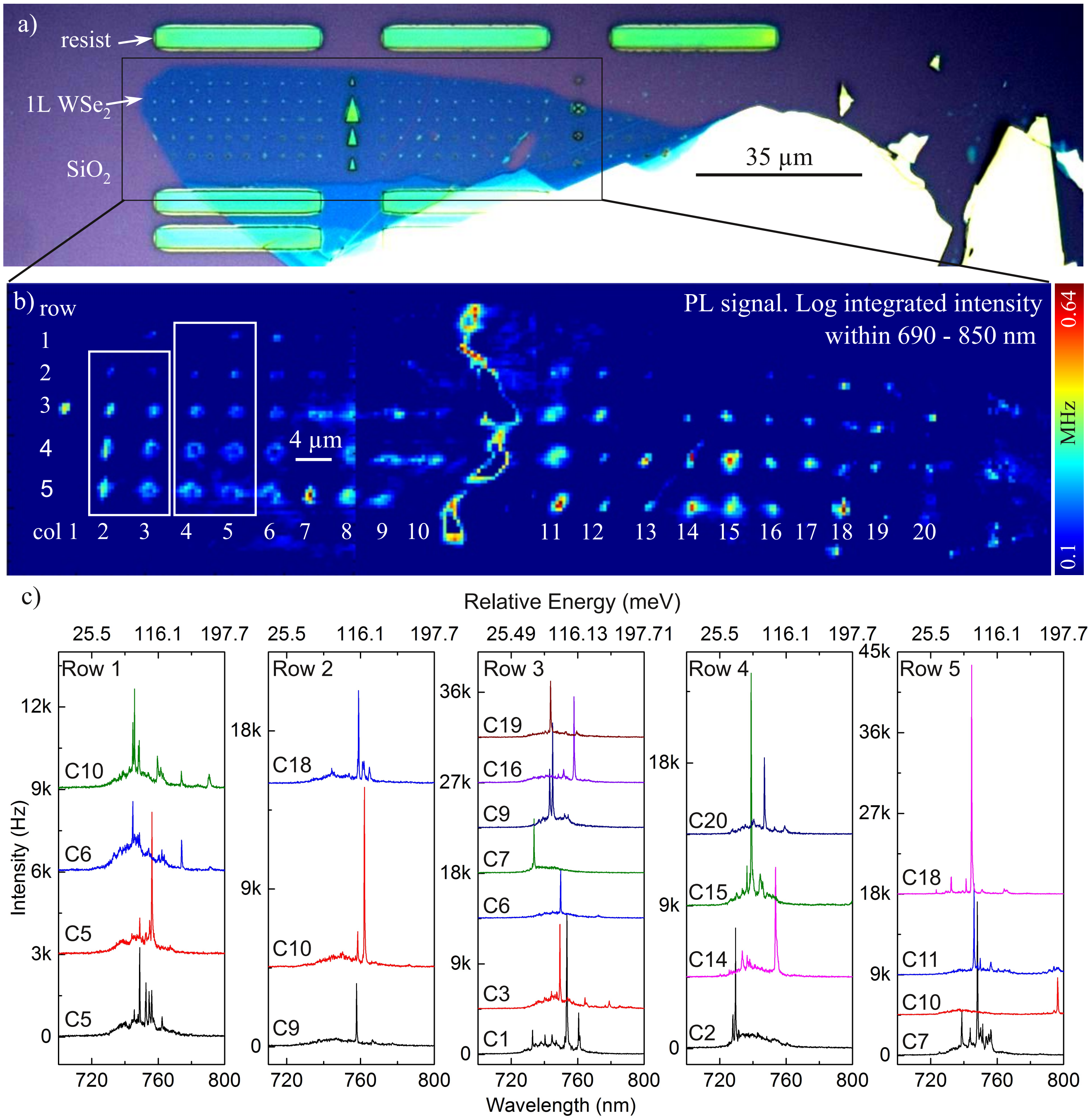

Supplementary Figure 4. Photoluminescence of strain-induced quantum emitters. (a) Optical micrograph of the flake of WSe_2 shown in Fig. 3 of the main article. (b) Spatial map of integrated PL signal showing the complete flake. The full map has 4 separate maps stitched together. During the second map, some motor hysteresis is visible. (c) PL spectra of individual emitters. The nanopillar position is indicated by a row-column reference (C = column).

Supplementary Note 5. In-depth optical characterization of monolayer WSe_2 emitter.

Supplementary Figure 5 shows the results from a time-trace measurement from the emitter at nanopillar #2 in the 1L flake from Fig. 1 in the main text. The left panel is a PL emission spectrum presenting a fine structure-splitting of 600 μeV. This spectrum is part of a time-trace measurement of 82 min with 5 s time bins. The center graph is an energy histogram integrated from the time-trace. The PL peak energy fluctuates 60 μeV. Finally, the right panel is an intensity histogram showing the non-blinking behaviour, with a mean intensity of 7000 counts per time bin.

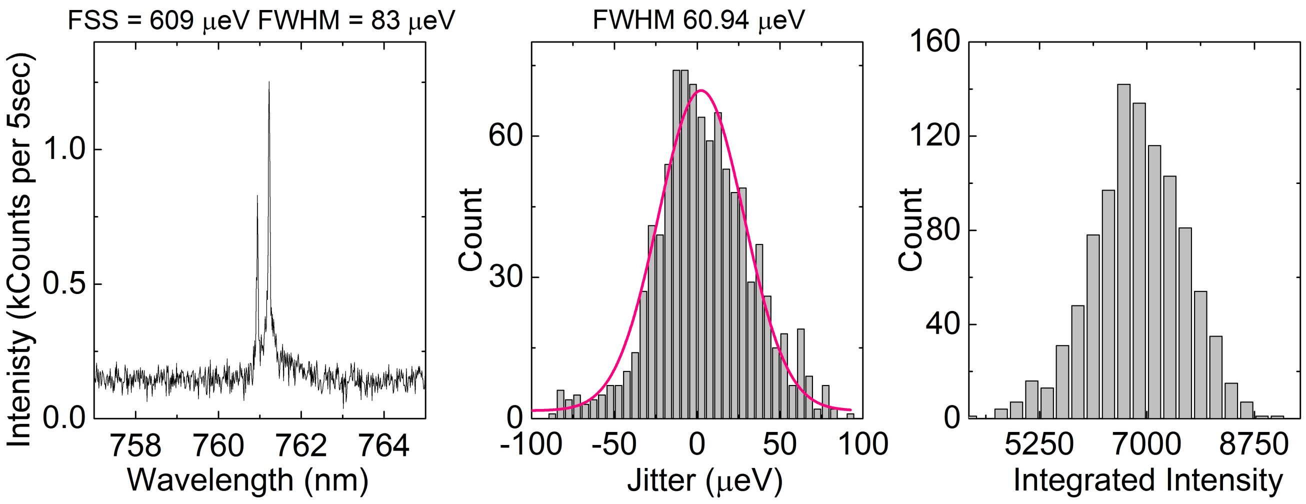

Supplementary Figure 5. Further optical characterization of a monolayer WSe_2 emitter. Left: Photoluminescence spectrum showing fine-structure splitting, extracted from a time-trace measurement. Center: Histogram of detuning for the time-trace measurement (82 min mea-surement with 5 s time bins). Right: Histogram of the integrated intensity showing PL stability during the time-trace measurement.
