# Supplementary material for: Deterministic strain-induced arrays of quantum emitters in a two-dimensional semiconductor
Source: Nat Commun. 2017 May 22;8:15053. doi: 10.1038/ncomms15053 (PMC5458118; doi:10.1038/ncomms15053)
Supplement: Peer Review File [file ncomms15053-s2.pdf]

Editorial Note: this manuscript has been previously reviewed at another journal that is not operating a transparent peer review scheme. This document only contains reviewer comments and rebuttal letters for versions considered at Nature Communications.
